# Relationship between loci of control and health-promoting behaviors in Pakistani women with polycystic ovary syndrome: coping strategies as mediators

**DOI:** 10.1186/s12905-021-01489-w

**Published:** 2021-10-09

**Authors:** Iram Fatima, Sahar Yaqoob, Farhat Jamil, Amna Butt

**Affiliations:** 1 Institute of Applied Psychology, University of the Punjab, Canal Road, Quaid‐e‐Azam Campus, Lahore, Pakistan; 2 Institute of Psychology, Beaconhouse National University, Lahore, Pakistan

**Keywords:** Polycystic ovary syndrome, Locus of control, Coping strategies, Health-promoting lifestyle behaviors

## Abstract

**Background/Objectives:**

Understanding the factors that promote healthy lifestyle behaviors in women with polycystic ovary syndrome is of substantial importance. Health-promoting lifestyle behaviors (HPLB) have been observed to be effective in managing various symptoms related to PCOS. This study aimed to examine the relationship between loci of control and health-promoting lifestyle behaviors in Pakistani women with polycystic ovary syndrome and the mediating role of coping strategies.

**Method:**

A correlational study was carried out with 145 unmarried women with polycystic ovary syndrome diagnosed by a gynecologist using the Rotterdam Criteria of 2003 (*M* age = 24.75 years). Participants were recruited from public sector hospitals in Lahore, Punjab, Pakistan and a series of hierarchical regression analyses were used to analyze results.

**Results:**

Findings suggest that women with internal and powerful others locus of control use more active practical coping strategies and less active distractive coping strategies. These women also get more involved in health-promoting behaviors. On the other hand, those with a high level of chance locus of control use less active practical coping strategies and more active distractive coping strategies. In turn, they engage less in health-promoting behaviors.

**Conclusion:**

Health professionals should consider the effects of different types of locus of control and coping strategies when planning interventions for women with polycystic ovary syndrome.

## Background

When women reach reproductive age, they face many challenges regarding health and well-being. They are more stigmatized than men if they are obese or are diagnosed with a reproductive disease. Polycystic ovary syndrome (PCOS) is defined as a range of symptoms, including ovarian cysts, irregular menstrual periods, excess growth of hair on the face, scalp thinning, acne, and weight gain [1]. In PCOS, the type and severity of symptoms can vary from woman to woman [2]. Following the Rotterdam Criteria of 2003, a diagnosis of PCOS is made when two out of three main characteristics, which include polycystic ovaries, high levels of androgens, and anovulation, are identified [3]. Women with PCOS are at risk of infertility [4], cardiovascular disease [5], type 2 diabetes, depression, and poor health [6]. Some of the challenges faced by women with chronic illness are poor nutrition, extreme inequity [4], and insufficient emotional and social support in the face of psychological distress [7].

The prevalence of PCOS varies around the world from 1.6% in women in the United States to 52% in women in India [8]. Although the prevalence of PCOS in Pakistani women is not known, one study has reported that around 54% of women visiting a hospital for gynecological issues suffer from PCOS [9]. Furthermore, it is more prevalent in women aged 15–30 years than in women aged over 30 years [10]. The modern lifestyle, which involves less physical activity, unhealthy dietary habits, gender role changes, and increased psychological pressures, is generally blamed for the increasing rate of PCOS in women in South Asia [11] and European countries [12]. Therefore, in recent years, behavioral management of PCOS has become a first-line treatment strategy [13, 14].

Health-promoting lifestyle behaviors (HPLB) have been observed to be effective in managing various symptoms related to PCOS. Structured exercise, weight loss, and cessation of smoking can reduce metabolic problems, including androgen imbalance [3, 15]. Behavioral intervention studies have shown that engaging in healthy eating and exercise has been linked to a reduction in menstrual issues, acne, and excess growth of body hair associated with PCOS [16]. A recent longitudinal study concluded that women with and without PCOS differ significantly in their lifestyles [17]. Despite growing evidence of the benefits of healthy behaviors and the advice of healthcare providers, sufferers, in general, find it difficult to make changes in their lifestyles [18]. Two important psychological factors are their locus of control and coping strategies, which can either support or hinder the adoption of health-promoting behaviors.

A person’s health locus of control (HLOC) can be described as how his or her internal and external sense of control helps to prevent and combat disease and involves both internal and external factors. Internal HLOC can be described as the belief that one’s own decisions and behaviors are responsible for one’s health. External HLOC is further divided into chance and powerful others HLOC. Chance HLOC is the belief that one’s health is determined by luck. Powerful others HLOC refers to dependency on others for one’s health [19, 20]. One meta-analysis revealed that internal HLOC, in general, is beneficial for health. However, internal HLOC and powerful others HLOC are related to specific health behaviors, depending on culture, age, and gender [21]. Chance HLOC, however, was not found to be related to any health behavior. More recent studies have also produced similar findings. For example, in studies conducted in the United States, it was found that people who rely on internal HLOC engage more in health-promoting behaviors, whereas those with chance HLOC indulge in less healthy lifestyle behaviors [22]. Similarly, in a longitudinal study in Australia, internal HLOC was found to play an important role in physical and mental health [23]. In other cross-sectional studies conducted in Iran [24], and Turkey [25], it was observed that powerful others HLOC and internal HLOC positively predicted health-related behaviors, whereas chance HLOC negatively predicted behaviors that promote health.

Another related construct benefitting people going through physical or psychological distress is coping strategy. Coping can be described as the effortful and purposeful use of strategies to manage specific stressful situations and to control distress related to stressful situations. The use of problem-focused coping strategies has been reported to be effective in the management of many symptoms related to PCOS in Iran [26] and in the United States [27]. Furthermore, it has been observed that individuals who have a high internal locus of control use practical coping strategies to improve their health [28]. One Chinese study demonstrated that confrontation coping strategies that involve practical efforts to confront and combat disease mediate the relationship between internal locus of control and healthy behaviors [29]

Overall findings from both Eastern and Western countries reveal a consistent positive role of internal HLOC and active practical coping strategies in health-promoting behaviors related to diet control and exercise, whereas findings from Eastern studies suggest that interdependent cultures facilitate the beneficial role of powerful others HLOC in women with PCOS. Furthermore, few studies have examined how different types of HLOC affect various health-promoting behaviors through coping strategies. Therefore, this study examined the relationship between HLOC and HPLB in Pakistani women with PCOS and the mediating role of coping strategies. Consistent with studies conducted in Asian countries, this study’s hypotheses were as follows: (1) internal and powerful others loci of control would positively predict HPLB, whereas chance HLOC would negatively predict HPLB, (2) active practical coping strategies would positively predict HPLB, and (3) active practical coping would mediate the relationship between internal HLOC and HPLB. In addition, the role of other coping strategies apart from active practical coping was explored as mediators between multiple dimensions of HLOC and HPLB.

## Methods

### Participants

Data were collected from 145 women with PCOS recruited from public sector hospitals in Lahore, Punjab, Pakistan. Out of 15 public sector hospitals with gynecology departments, 7 agreed to participate in the study. A total of 182 unmarried women with the age range of 19–30 years (*M* = 24.57, *SD* = 2.38) diagnosed by a gynecologist using the Rotterdam Criteria of 2003 were approached. Of these, 34 refused to participate, and 3 did not understand the language of the questionnaire, and, therefore, the total response rate was 79.67%. Of all the participants, 83% of the women reported having PCOS from 2 to 4 years and 17% from 5 to 7 years. Ninety-six percent were obese, and 4% were overweight, as determined by their body mass index.

### Measures

*Multidimensional health locus of control Scale *[30] was used to assess health-related loci of control. The scale consists of three subscales: Internal HLOC, Chance HLOC, and Powerful others. Responses are based on a five-point scale from 1 (strongly disagree) to 5 (strongly agree). Cronbach’s alpha coefficients of the three subscales were 0.88, 0.74, and 0.74, respectively, for the present study.

*Coping strategies questionnaire* [31] consists of four subscales: Active Practical Coping Scale, Religious Focused Coping Scale, Active Distractive Scale, and Avoidance Scale. Responses are based on a 5-point scale ranging from 1 (“does not apply”) to 5 (“extremely applicable”). The questionnaire was initially developed in Urdu for use in Pakistan. Cronbach’s alpha coefficients of the subscales were 0.91, 0.53, 0.80, and 0.78, respectively, for the present study.

*Health-promoting lifestyle profile II, adult version* [15] was used to assess behaviors involved in promoting health. This profile consists of six subscales: Physical Activity, Nutrition, Interpersonal Relations, Spiritual Growth, Health Responsibility, and Stress Management. Responses are based on a 4-point Likert scale ranging from 1 (“never”) to 4 (“routinely”). Cronbach’s alpha coefficients of the six subscales for the present study were 0.85, 0.84, 0.82, 0.85, 0.85, and 0.84, respectively.

All questionnaires were administered in the national language Urdu. Scaled scores were calculated for each scale. A higher score indicated a higher endorsement of the construct.

### Procedure

After ethical approval from the Departmental committee and formal permission from hospital administrators, the first author contacted the outpatient gynecologists and informed them about the study. At a time provided by the women’s doctors, the researcher met with participants in a separate designated area. The purpose of the study was explained to the participants, and prior verbal and written informed consent was obtained from each. Those who agreed to participate were given a set of questionnaires and were asked to respond by marking the appropriate response after reading each item carefully. The questionnaires took a total of 25 to 35 min to complete.

### Data analysis

Pearson product-moment correlation analysis was used to assess the relationship among study variables. To test if coping strategies mediate between different aspects of HLOC and HPLB, two sets of multiple hierarchical regression analyses with the enter method were carried out. The first set of analyses was carried out with the dimensions of HLOC in block one and the coping strategies in block 2, with the dimensions of HPLB treated as separate dependent variables. The direct effects of different dimensions of HLOC and coping strategies were observed from the second block, and the total effects of different dimensions of HLOC were measured from the first block for the different dimensions of HPLB.

In the second series of analyses, linear regression analyses with the enter method were carried out with internal, powerful others, and chance HLOC in block 1 and the coping strategies as dependent variables. The direct effects of the different dimensions of HLOC on coping strategies were measured. Sobel Z test was conducted to test for significance of indirect effects.

## Results

Table 1 shows findings of Pearson’s correlations and it can be observed that internal and powerful others HLOC had significant positive correlations with HPLB, whereas chance HLOC had significant negative correlations with HPLB and its aspects. Among the dimensions of coping strategies, active practical coping had significant positive correlations with HPLB and its dimensions. Whereas active distractive and avoidance-focused coping had significant negative correlations with HPLB and its aspects. Further, a significant negative correlation of active practical coping strategy was observed with avoidance and active distractive coping strategies.

**Table 1:** Pearson’s correlation coefficients among locus of control, coping strategies, and health-promoting lifestyle behaviors

Variable	2	3	4	5	6	7	8	9	10	11	12	13	*M*	*SD*
1. Internal HLOC	− .01	.19*	.31***	− .04	− .19*	− .26**	.41***	.39***	.40***	.41***	.39***	.41***	6.37	2.36
2. Powerful others HLOC		− .05	.33***	.17*	− .31***	− .28**	.25**	.22**	.25**	.21**	.27**	.27**	4.34	1.36
3. Chance HLOC			− .39***	− .09	.28**	.24**	− .34***	− .35***	− .33***	− .33***	− .34***	− .35***	5.25	1.59
4. Active practical CS				.05	− .57***	− .59***	.72***	.69***	.71***	.69***	.70***	.71***	3.79	1.01
5. Religious focused CS						.25**	.28**	.05	− .00	.00	.05	.06	5.94	1.02
6. Avoidance focused CS						.76 ***	− .44***	− .44***	− .44***	− .44***	− .46***	− .45***	5.52	1.12
7. Active distractive CS							− .51***	− .52***	− .50***	− .49***	− .47***	− .52***	5.29	1.49
8. Health responsibility								.94***	.95***	.93***	.92***	.94***	7.68	2.08
9. Physical activity									.92***	.9 1***	.89***	.95***	7.63	2.18
10. Nutrition										.93***	.90***	.94***	7.69	2.00
11. Spiritual growth											.91***	.94***	7.72	2.06
12. Interpersonal relations												.92***	7.64	1.97
13. Stress management													7.69	2.07

*CS* coping strategy, *HLOC* health locus of control

*p < .05; **p < .01; ***p < .001

Internal HLOC had a significant positive correlation with active practical coping and a significant negative correlation with avoidant and distractive coping strategies. Powerful others HLOC had a significant positive correlation with active practical coping and religious coping whereas it showed a significant negative correlation with avoidant and distractive coping strategies. Chance HLOC had a significant negative correlation with active practical coping and a significant positive correlation with avoidant and distractive coping strategies.

Results from hierarchical linear regression analyses with six dimensions of HPLB as criteria are presented in Table 2 It can be seen that total and direct effects of internal HLOC on six dimensions of HPLB were positive and significant, although, the strength of direct effects was less than total effects. Further, total effects of powerful others HLOC were positive and significant for all HPLB dimensions while the direct effects were not significant. Total and direct effects of chance HLOC on all dimensions of HPLB were negative and significant, although, the strength of direct effects was weaker. Among the coping strategies, active practical coping positively predicted all dimensions of HPLB. In addition, active distractive coping negatively predicted physical activity, health responsibility, and stress management.

**Table 2:** Unstandardized regression coefficients for direct and total effects of HLOC and coping strategies on HPLB

Variables	Physical activity		Nutrition		Spiritual growth		Health responsibility		Interpersonal relations		Stress management	
	Direct	Total	Direct	Total	Direct	Total	Direct	Total	Direct	Total	Direct	Total
Internal HLOC	.32***	.45***	.28***	.40***	.31***	.44 ***	.30***	.43***	.29***	.40***	.33***	.44***
Chance HLOC	− .38***	− .56***	− .30**	− .48***	− .27**	− .47***	− .31**	− .50***	− .29**	− .47***	− .35***	− .52***
Powerful others HLOC	.04	.20*	.09	.23**	.00	.18*	.08	.26**	.10	.27**	.12	.27**
Active practical CS	.32**		.32***		.32**		.33***		.28**		.27**	
Active distractive CS	− .32*		− .21		− .20		− .24*		− .06		− .25*	
Avoidance focused CS	.27		.20		.09		.19		.04		.19	
Religious focused CS	.03		.00		.15		.11		.13		.10	

*CS* coping strategy, *HLOC* health locus of control, *HPLB *health-promoting lifestyle behaviors

*p < .05; **p < .01; ***p < .001

Results from multiple linear regression analysis with coping strategies as criteria are given in Table 3. Internal HLOC and Powerful others HLOC positively predicted active practical coping. Besides, they negatively predicted active distractive coping and avoidance-focused coping. In addition powerful others HLOC also positively predicted religious focused coping. Further, Table 3 indicates that chance HLOC negatively predicted active practical coping and positively predicted active distractive coping and avoidance focused coping. The results in Tables 2 and 3 suggest that active practical coping and active distractive coping mediated between HLOC and HPLB following the approach of Baron and Keney [32]

**Table 3:** Unstandardized regression coefficients for direct effects of HLOC on coping strategies

Variables	Coping strategies			
	Active practical	Active distractive	Avoidance focused	Religious focused
Internal HLOC	.35***	− .20***	− .12**	− .01
Chance HLOC	− .58***	.27***	.22***	− .05
Powerful others HLOC	.47***	− .30***	− .24***	.13*

*HLOC* health locus of control

*p < .05; **p < .01; ***p < .001

To test the mediation hypothesis further, the Series of Sobel’s Z test was applied. The results confirmed that active practical coping mediated the relationships between internal HLOC and physical activity, nutrition, spiritual growth, health responsibility, interpersonal relations, and stress management, Z = 3.91, 3.94, 3.92, 3.94, 3.91, and 3.93, respectively, *p* < 0.001. The Sobel Z test also confirmed the finding that active distractive coping mediated the relationships of internal HLOC with physical activity, health responsibility, and stress management dimensions of HPLB, Z = 2.99, 2.96, and 2.98, respectively, *p* < 0.001.

It was also observed that active practical coping mediated the relationships between powerful others HLOC and physical activity, nutrition, spiritual growth, health responsibility, interpersonal relations, and stress management, Z = 3.74, 3.79, 3.74, 3.78, 3.75, and 3.77, respectively, *p* < 0.001. Active distractive coping mediated the relationships between powerful others HLOC and physical activity, health responsibility, and stress management dimensions of HPLB, Z = 3.16, 3.12, and 3.13, respectively, *p* < 0.001.

The Sobel’s Z-test also supported that active practical coping mediated the relationships between chance HLOC and physical activity, nutrition, spiritual growth, health responsibility, interpersonal relations, and stress management, Z = − 4.59, − 4.65, − 4.61, − 4.61, − 4.67, − 4.61, and − 4.62, respectively, *p* < 0.001. It was confirmed by applying Sobel’s Z-test that active distractive coping mediated the relationships of chance HLOC with physical activity, health responsibility, and stress management dimensions of HPLB, Z = − 2.88, − 2.85, and − 2.87, respectively, *p* < 0.001.

Overall, the results suggest that women with polycystic ovary syndrome with high internal and powerful others HLOC use more active practical coping strategies, which enable them to engage in the different forms of health-promoting behaviors. In contrast, high levels of chance HLOC hamper active practical coping, which further leads to less involvement in the dimensions of HPLB. Besides, high internal and powerful others HLOC and low levels of chance HLOC lead to less active distractive coping strategies, which in turn lead to more engagement in some of the health-promoting behaviors.

## Discussion

This study was conducted to assess the correlations between three dimensions of HLOC (internal, chance, and powerful others) and health-promoting lifestyle behaviors in Pakistani women with PCOS. It further aimed to explore whether coping strategies mediated these correlations. It was observed that both internal and powerful others HLOC positively predicted aspects of health-promoting behaviors. This finding was in line with those of studies conducted in Asian countries, Iran [24] and Turkey [25]. In general, the belief that one is in charge of one’s own life facilitates engagement in self-benefitting acts. However, studies conducted in individualistic cultures don’t support the adaptive role of Powerful others locus of control [21–23]. Trust in the judgment and direction of others in charge has been found to be motivating in societies and groups where people typically accept the control of those in power, as in collectivistic cultures [21]. Pakistan is a country with a collectivistic culture, where people rely on others such as friends and other significant people in their lives for their well-being, and the opinion of health experts becomes particularly important when facing an illness.

It was also found that active practical coping strategies predicted health-promoting behaviors. This finding was similar to those from the United States [27] and in Iran [26]. Active practical coping involves awareness of problems and the adoption of strategies to solve them, and a health-related problem demands both knowledge about the problem and engagement in behaviors that are known to mitigate the problem.

Despite knowing what is beneficial and what is not, some people still do not engage in health-promoting behaviors. An important reason for involvement in active goal-directed activities may be a person’s locus of control [28, 29]. It was observed that women with an internal locus of control used more active practical coping strategies, which in turn led to more use of health-promoting behaviors. The belief that one can bring about changes in one’s life leads to the adoption of more goal-oriented strategies, which further leads to more engagement in health-oriented behaviors when faced with health-related problems.

Active practical coping strategies also mediate between the powerful others locus of control and health-promoting behaviors in women with PCOS. Pakistan is a collectivistic society in which a person’s self-worth, especially for women, is to a great extent dependent on others. For women who seek approval from society, PCOS is a condition that is stigmatizing, as it is associated with infertility, obesity, and excess growth of body hair [33]. For these women, advice from more knowledgeable people may motivate them to actively address their condition and socially undesirable symptoms even when they would prefer to not do so [21].

It was also observed that active distractive coping strategy negatively predicted some health-promoting behaviors. It also acted as a mediator in the correlations between the three types of HLOC and health-promoting behaviors. Distraction has been shown to help reduce pain and stress that are not in one’s control [34]. However, for situations that require active solutions, such strategies are ineffective. Polycystic ovary syndrome is characterized by many physical symptoms that can worsen if ignored [11, 12].

Furthermore, chance HLOC negatively predicted health-promoting behaviors through less active practical and more distractive coping strategies. Earlier studies have also demonstrated the negative role of chance HLOC in taking practical initiatives to improve one’s life [21, 22, 24, 25]. People who believe that their situation is determined by random events are less likely to take active measures to change their lives. They instead use distractive strategies to reduce pain caused by illnesses, which can provide short-term relief but do not offer long-term benefits.

The study has a few limitations. Assessments were made through self-report measures. Although healthy behaviors are measured conventionally this way. They could be measured more objectively through other ways which could include monitoring physical exercises, spiritual growth, stress management, and health responsible behaviors over a given course of time. Another limitation of the study is that the correlational design of the study prevents us from drawing the casual relations and checking whether the locus of control causes any changes in coping and health-promoting behaviors. So it is suggested to use a longitudinal research design to study these variables.

## Conclusion

It can be concluded that both having belief in one’s self and relying on others regarding one’s health can motivate one to use more active practical strategies and less active distractive strategies, which in turn leads to the use of more health-promoting lifestyle behaviors. However, believing that health issues are unmanageable and determined by chance factors negatively affects engagement in health-promoting activities, through less use of active practical coping strategies and more use of active distractive coping strategies.

This study has important implications for health professionals. They should consider the locus of control and coping strategies when aiming to enhance health-promoting lifestyle behaviors in women with PCOS. Interventions should be based on the control-related beliefs and coping strategies generally used by their patients. It is recommended that health professionals should psycho-educate women about the role of internal locus of control in determining their health-related choices. Women should be encouraged to make active and helpful health choices when making decisions to manage polycystic ovary syndrome (PCOS). Women should be given guidelines to promote regular exercise, good nutritional intake, and effective stress management. In addition, they should be encouraged to involve the family in helping them to address PCOS. Moreover, health care professionals can also encourage families of women with PCOS to closely monitor their health-related decisions and give them timely constructive feedback to address the concern. Health care professionals can also make referrals to mental health professionals if unhealthy choices and avoidant strategies are identified to be risk factors for distress in women. Similarly, timely consultation with nutritionists should also be suggested to the women who seem to be struggling with unhealthy food choices and obesity despite the intervention of gynecologists.

## Data Availability

Data are available from the corresponding author on reasonable request.

## Abbreviations

PCOS: Polycystic ovary syndrome
HPLB: Health-promoting lifestyle behaviors
HLOC: Health locus of control
